# Navigating the evolution: a comprehensive review of sustainable finance in mergers and acquisitions

**DOI:** 10.12688/f1000research.150207.1

**Published:** 2025-03-14

**Authors:** Sabyasachi Mondal, Debdas Rakshit, Kishan Jee, Satish Chandra Tiwari

**Affiliations:** 1 Department of Commerce, The University of Burdwan, Bardhaman, West Bengal, India; 2 Brainware University, Kolkata, West Bengal, India; 3 Tezpur University, Napaam, Assam, India

**Keywords:** Sustainable Finance, Mergers and Acquisitions, Big Data Analytics and Machine Learning, Green Bonds and Artificial Intelligence, Systematic Review

## Abstract

**Background:**

The global shift in corporate philosophy from wealth maximization to share value creation has propelled sustainable finance into the forefront of research endeavours. Despite its significance, the realm of sustainable finance, especially in the context of mergers and acquisitions, remains relatively underexplored.

**Objective:**

This research intends to identify the gap by conducting a cutting-edge and expansive systematic review, unravelling the intricate layers of sustainable finance concerning mergers and acquisitions.

**Data source:**

The data source is Web of Science to start with, yielding 41 publications. A similar search on Scopus yielded 52 articles. Not surprisingly, the 41 publications discovered via the Web of Science search were also found in the Scopus results.

**Study eligibility criteria:**

Papers on business, management, environmental studies, corporate finance, economics, and ethics are featured.

**Study appraisal and synthesis methods:**

Employing advanced methodologies including machine learning tools, our research delves into domain specific influential papers, top-contributing journals, noteworthy authors, institutions, and nations. By dissecting the context and methodologies employed in scholarly works, we aim to instil a through overview of the current body of knowledge. our study categorizes the selected scholarly research into four distinct objectives, facilitating a nuanced understanding of the diverse perspectives within the field. Each stratum of papers is meticulously analysed and discussed, offering insights into key themes and patterns. To guide future research initiatives, we present a synthesis of pertinent research questions, exploring avenues such as the incorporation of innovative financial instruments like green bonds and artificial intelligence.

**Results:**

Since the Sustainable Development Goals were introduced in 2016, sustainable finance has become an increasingly important topic in merger and acquisition research. Research is progressing at a rapid pace in China and Australia, while emerging nations are falling behind. In addition, this study specifies the necessity of conducting a more comprehensive examination of the habits of company representatives regarding sustainable investments. This aspect has been neglected in earlier research. Additionally, because global M&As are becoming more common, research on cross-border M&As must take precedence over domestic ones. Finally, there is a gap in the current body of knowledge about greenwashing, green audits, and the use of AI in sustainable finance.

**Conclusions and implications of key findings:**

Machine learning and big data analytics may help scholars get a more thorough grasp of the historical and present significance of sustainability in M&A. New era researchers may advance the potential yet unexplored topics.

## 1. Introduction

Over the course of recent years, there has been a noticeable transformation in corporate philosophy, shifting from the traditional emphasis on profit maximization, as articulated by Friedman in 1962, to a contemporary ethos centered around shared value creation, as expounded by Kramer and Porter in 2011. This evolution has prompted managerial engagement of various stakeholders, leading to the creation of sustainability as a multifaceted construct encompassing environmental, social, and governance (ESG) considerations, as posited by Orlitzky et al. (2003). This has resulted in the notion of sustainable finance, which incorporates ESG considerations into the finance driven decision-making processes of a firm ( Edmans &Kacperczyk, 2022).

Stakeholders, on their part, have started making companies accountable for their dealings in environmental, social and governmental issues ( Galbreath, 2013). The aftermath of the 2008 financial crisis has further intensified this trend. Moreover, pressure form supranational entities such as United Nations and European Union ( Kaspereit & Lopatta, 2016), influence from shareholders ( Boerner, 2010; Manescu et al., 2011), and influence of NGOs ( OECD, 2014), have propelled the companies to redesign their strategies to remain competitive by trading off between profit motive and sustainability thrust. 

United Nations initiatives on sustainability are two-pronged. One is a yearly convention held for climate change ( COP, 2021) and another shared blueprints on sustainability on pre-decided objectives and their implementation (SDGs). Sustainable Development Goals (SDGs) were designed in 2015 to cater to 17 objectives for a prosperous and peaceful future to be implemented by 2030 ( United Nations, 2015). An estimated investment of around $5 to $7 trillion is estimated to occur to achieve the SDGs ( COP, 2021). Moreover, the Covid-19 pandemic have reinforced the idea of sustainability among corporates. Therefore, corporate substitutability is a relevant topic among researchers and corporates alike.

The main purpose of a corporate entity is to create value for the shareholders. One of the ways to create value is through M&A. There are two contrasting theories related to M&A namely stakeholders’ theory ( Freeman et al., 2004) and the Shareholders’ Theory ( Friedman, 2007; E. Chen & Gavious, 2015).

Stakeholders’ Theory, as articulated by influential scholars ( Ferrell et al., 2016), posits that the firm has three motives namely instrumental (driven by self-interest), relational (driven by relationship among the stakeholders) and moral (driven by moral principles and ethical standards) ( Aguilera et al., 2007). Stakeholder theory suggests that socially responsible actions including ample employee benefits, sick leaves, cost control and thereby the final price control can fetch good reputation from the stakeholders. Reputation reflects positively on customer loyalty ( Walsh et al., 2019), trust, and identification ( Keh & Xie, 2009) and these reflections catapults customers’ willingness to pay ( Homburg et al., 2005). 

Consequently, the revenue and profit outlook improve which reflects positively on the share price. On the other hand, shareholder’s theory proclaims that the only goal of business is to utilize assets to the maximum. If the CSR activities are done consistently, it will take away a chunk of resources which could have been utilized for some productive activities ( Friedman, 2007).This apparent dichotomy between the two streams of thoughts has necessitated a detailed analysis of the seminal works in these areas.

The apparent tension between these two streams of thought necessitates a comprehensive analysis of seminal works in these areas. This research aims to bridge this theoretical gap, providing insights into the implications of M&A activities for value creation in the context of divergent stakeholder and shareholder perspectives.

In this research, we intend to provide an up-to-date overview of sustainable finance researches in view of M&A. Bibliometric analysis is used for the study which suits reviewing large set of journal articles using sophisticated quantitative measures ( Donthu et al., 2021) is used in this study. Bibliometric analysis highlights the application of machine learning on scholarly researches in two different ways namely,

Searching of Data: The study for the scholarly researches is conducted in Web of Science (WOS) database using specified keywords for supervised machine learning to extract a large amount of bibliometric data relating to relevant articles on sustainable finance related to M&A ( Donthu et al., 2021).

Analysis of Data: EThe multi-faceted (e.g., keywords, journals, authors, institutions, countries), multi formatted (e.g., words), and in a large scale (322 articles) analysis are guided by unsupervised machine learning, unearthing interrelated keywords and the identical clusters of major themes ( Kumar et al., 2021).

The central thematic inquiry of the paper revolves around the value creation for shareholders through the incorporation of sustainability considerations in M&A decisions. This inquiry is meticulously addressed through a synthesis of existing research via systematic review and content analysis. Subsequently, the paper not only encapsulates the current state of scholarship but also provides a trajectory for future research directions.

The systematic literature review and bibliometric analysis contribute significantly to the enrichment of sustainability and M&A research, as evidenced by the formulation of six pertinent research questions ( Donthu et al., 2021)

RQ1: What are the prevailing publication trends characterizing sustainable finance research within the context of Mergers and Acquisitions (M&A)?

RQ2: Which journals exhibit the highest impact and contribute significantly to the field of sustainable finance research related to M&A, and most influential articles within these journals?

RQ3: Who are the prominent contributors in terms of geography, institution and authors, shaping the landscape of sustainable finance research in the realm of M&A?

RQ4: identify the methodological choices are prevalent, and what research contexts are identified in the current body of sustainable finance research concerning M&A?

RQ5: What overarching themes and specific topics emerge as central within the domain of sustainable finance research in the context of M&A?

RQ6: What future research directions can be discerned for sustainable finance within the framework of M&A, including potential areas of exploration such as the integration of innovative financial instruments like green bonds and artificial intelligence?

The comprehensive literature review undertaken in this study yields multifaceted insights crucial for the advancement of research in sustainable finance within the context of M&A. Firstly, the analysis of publication trends addresses Research Question 1 (RQ1), providing new-age researchers with a nuanced understanding of the historical progression of scientific inquiries in this domain. Secondly, researchers of this area will know the potential journals for publication and can also identify key literature (RQ2). Thirdly, researchers will find potential institutions or authors to collaborate (RQ3). Fourthly, potential researchers will get insight into the methodologies already adopted (RQ4). Fifthly, researchers will be aware of the major themes related to this area. This will help them work in the areas hitherto untouched (RQ5). Lastly, researchers will be encouraged to chart new path in this area taking inspiration from the past research (RQ6). The review will not only help companies include sustainability, be it environmental, social or governmental in their regular affair to enhance value in pre- or post-merger condition, it will guide the shareholders exert pressure on the management to include sustainability as a tool to enhance value. Moreover, the policymakers will be encouraged to influence investors to invest in firms which are sustainable in nature either through direct investment through shares or through socially responsible indices (SRIs). Lastly, the review catalyzes charting new paths in sustainable finance. By inspiring researchers to draw inspiration from past studies (RQ6), this analysis encourages the formulation of novel research questions and methodologies, driving continuous progress in the field.

Other than its academic significance, this review provides practical insights for companies, shareholders, and policymakers. It propels the companies to incorporate sustainability, be it environmental, social, or governmental, into regular business practices to improve value in both pre-and post-merger conditions. Moreover, it provides shareholders with the tools to exert pressure on management, emphasizing the role of sustainability as a value-enhancing tool. Policymakers, in turn, are encouraged to influence investors to direct their investments towards sustainable firms, whether through direct share investment or participation in socially responsible indices (SRIs). In sum, the outcomes of this literature review extend beyond academia, offering valuable insights with the potential to shape corporate practices, investor behavior, and policymaking in the realm of sustainable finance and mergers and acquisitions.

## 2. Concept of sustainable finance

The prominence of sustainability in financial discourse has notably increased since the 2008 financial crisis, largely attributed to the ethical lapses in corporate practices within the USA ( Ökte, 2012). The foundational work of ( Ferris & Rykaczewski, 1986) marked the early exploration of social investing’s utility in portfolio management, laying the groundwork for subsequent literature on sustainable finance ( Ferrell et al., 2016).

Terminologies associated with sustainable finance exhibit a spectrum of nuances, with (review & 1999, n.d.) highlighting the subjectivity in the expression of sustainability ( Dorfleitner & Utz, 2012) succinctly encapsulate sustainable investments as encompassing all non-financial impacts. Sandberg et al. (2009) defines social responsibility as the integration of non-financial concerns, including ethical, social, and environmental aspects, into the investment process.

Literature reviews abound on various facets of sustainable finance, such as environmental, social, and governmental (ESG) considerations ( Daugaard, 2020), green finance (L. Chen et al., 2021), climate finance ( Engle et al., 2020), impact investing ( Clarkin & L. Cangioni, 2016), and socially responsible investing ( Camilleri, 2021), each contributing to the broader understanding of sustainability.

Corporate social responsibility (CSR) gained traction in the corporate lexicon during the early 21st century. Hatten et al. (2020), aligning with ( Carroll & Shabana, 2010) classification of economic, legal, ethical, and philanthropic responsibilities. ( Kurtz, 2008) further categorized social responsibility investment (SRI) to encompass ethical, philanthropic, social, and environmental impacts. ( Ferrell et al., 2016) offered an overview of the growing trend of social responsibility investments across US, European, Canadian, and Australian markets, indicating an increasing interest in combining financial and ethical motives.

The literature indicates that sustainable practices contribute to enhancing corporate reputation ( Arouri et al., 2019), improving financial performance ( Eccles et al., 2014), influencing market value ( Kaspereit & Lopatta, 2016), facilitating better access and reducing the cost of equity financing ( El Ghoul et al., 2011), and mitigating risks ( Arouri et al., 2019).

## 3. Sustainability and M&A

The concept of sustainability has gained prominence post 2008 financial meltdown and the crisis was largely attributed to the downfall of corporate ethical practices across USA. However, the literature support was provided by Ferris and Rykaczewski (1986), who verified the utility of social investing in portfolio management. The terminologies related to sustainable finance are manifold. There are heterogeneities in the way the sustainability is uttered and it comes from matter of taste ( Cowton, 1999). Sustainable investments summarize every non-financial impact ( Dorfleitner and Utz, 2012). Sandberg et al. (2009, page number) proclaimed that social responsibility is an ‘‘integration of certain non-financial concerns, such as ethical, social or environmental, into the investment process.’’ There are ample literature reviews based on environmental, social and governmental (ESG), green finance, climate finance, impact investing, and socially responsible investing, all of which are different names attributed to sustainability ( Daugaard, 2020; Zhang, et al., 2019; Giglio et al., 2021; Clarkin & Cangioni, 2016; Camilleri, 2021; Rahman & Rahman, 2020; Viviers & Eccles, 2012).

The concept of corporate social responsibility (CSR) gradually made its way in the mainstream corporate lexicon in the beginning of 21st century ( Carroll, 1991). Corporate social responsibility (CSR) is floated namely economic, legal, ethical, and philanthropic responsibilities as suggested by Archie Carroll (1991). Likewise, Kurtz (2008) broadly classified social responsibility investment (SRI) as something that impacts ethically, philanthropically, socially and environmentally. SRI had finally come out in open from being practiced by a small group of specialist funds to the boardroom of most of the financial institutions ( Sparkes & Cowton, 2004).

Diverse theories explained the association between firms’ financial performance and ESG performance. Stakeholder theory talks about a positive relationship, trade-off theory portrays a negative relationship emphasizing the potential conflict of interest between financial performance and ethical considerations ( Qureshi et al., 2021) and slack resources theory posits a positive association and the managerial opportunism theory postulates a negative association ( Tanggamani et al., 2018). All these theories points to a bidirectional causal relation and leading to a ‘vicious cycle’ ( Wissink, 2012). However, the association between ESGP and CFP remains inconclusive ( Wang et al., 2016). The inconclusiveness in theoretical and empirical researches regarding the association between ESG and firms’ financial performance depicts a non-static form of relationship ( Brammer & Millington, 2008; Lu et al., 2014; Margolis & Walsh, 2003).

Furthermore, factors including corporate culture, employee knowledge, and reputations, are acknowledged as value generators for companies ( Wernerfelt, 1984). However, these factors are inherently challenging to quantify. In the context of M&A, organizational culture emerges as a significant financial driver. ESG scores act as a viable representative for firm culture in both acquiring and target companies. Notably, ESG compatibility has gained traction as a lens through which M&A transactions are evaluated, reflecting the increasing recognition of the pivotal role of cultural cohesion in these deals ( Alexandridis et al., 2022).

Studies by ( Morrow et al., 2017) demonstrates that M&A deals characterized by ESG compatibility outperform those that lack such alignment, achieving a cumulative average return of 21% over five years. While this observation does not offer a definitive conclusion, it suggests that companies with robust ESG profiles may experience fewer cultural frictions in the post-acquisition period, potentially resulting in superior financial returns compared to companies with inferior ESG profiles. Consequently, analyzing the post-acquisition financial performance of the acquirer in conjunction with the ESG scores of both the acquirer and the acquiree becomes imperative for a comprehensive understanding of the relationship between ESGP and M&A outcomes (C. Chen et al., 2022).

## 4. Methods

The systematic review conducted includes journal articles on business, management, environmental studies, corporate finance, economics, and ethics only. Moreover, duplicate records and reports relating to knowledge acquisition are not included. At the end, articles not related to M&A and where sustainability and M&A words are included as keyword are excluded from our analysis.

The bibliometric data on the sustainability and value creation through M&A is collected through Scientific Procedures and Rationales for Systematic Literature Reviews (SPAR-4-SLR) protocol, which consists of three stages namely assembling, arranging, and assessing of journal articles ( Donthu et al., 2021).

In the assembling phase, those scholarly articles were chosen which focuses on nexus between CSR and value creation in the context of M&A ( Caiazza et al., 2021). This involves a comprehensive search and retrieval process to identify pertinent publications.

The arranging stage, thereafter involves organized the literatures systematically. The identified articles are then categorized depending on the key parameters such as publication date, authorship, methodology, and thematic relevance ( Donthu et al., 2021). This paved way for a structured putline of the existing body of literature. 

The assessing stage entails a thorough evaluation of the amassed literature. The articles undergo a critical examination phase to ascertain its significance, methodological rigor, and contribution to the understanding of sustainability and value creation in M&A context. This assessment ensures that only high-quality and relevant studies are included in the subsequent analysis. 

**Figure 1.**
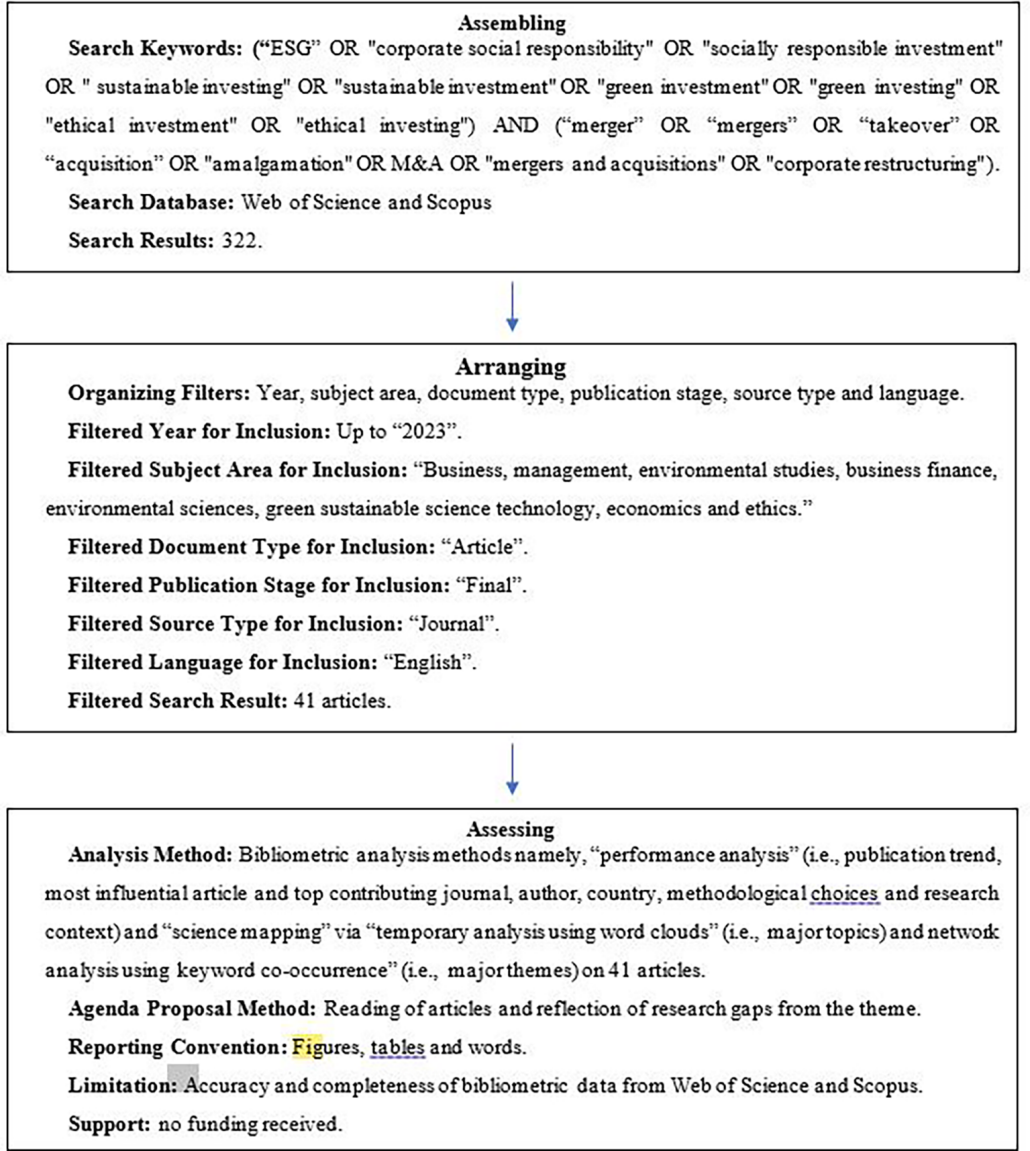
Systematic review procedure using the SPAR-4-SLR protocol.

By adhering to the SPAR-4-SLR protocol, this literature review ensures a systematic and rigorous approach to the bibliometric analysis, offering a comprehensive overview of the scholarly landscape surrounding sustainability and value creation through M&A ( Figure 1).

### 4.1 Assembling

To assemble the articles on effect of sustainability on value creation through M&A is done with the help of keywords. The keywords are searched with the help of past literatures and search word generation app from the library of University of Wollongong Australia. The keywords are found from the initial review of relevant literature, few of which are mentioned in the previous section. The search culminated to keywords those were connected into the following string: (“ESG” OR “corporate social responsibility” OR “socially responsible investment” OR “ sustainable investing” OR “sustainable investment” OR “green investment” OR “green investing” OR “ethical investment” OR “ethical investing") AND (“merger” OR “mergers” OR “takeover” OR “acquisition” OR “amalgamation” OR M&A OR “mergers and acquisitions” OR “corporate restructuring”).

### 4.2 Arranging

**Figure 2.**
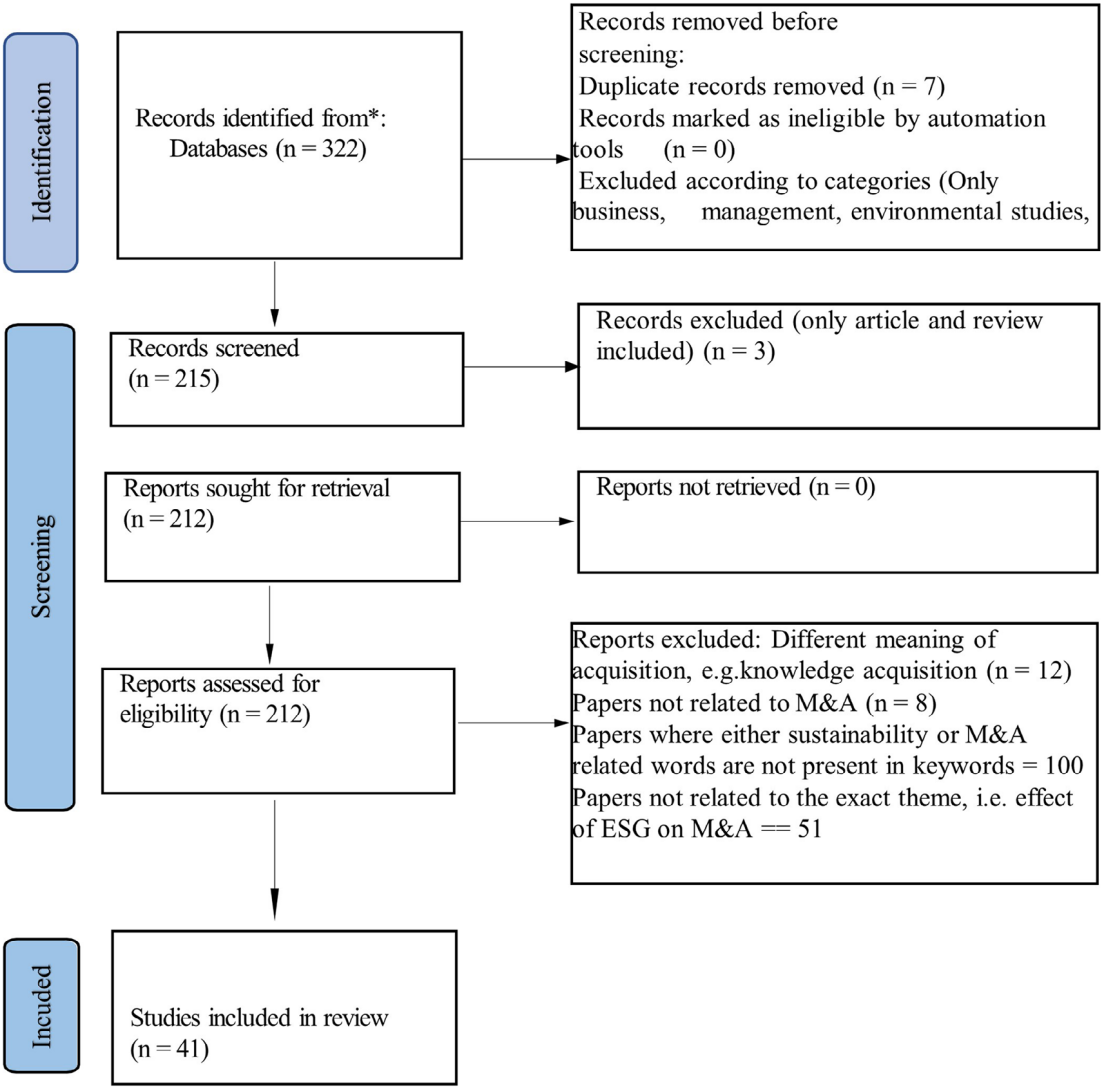
The PRISMA flow diagram (identification of studies via database).

Those keywords are used to find literature and downloaded in text or tab-delimited format. To reach the desired literature set, identification, screening, and inclusion processes are followed per Preferred Reporting Items for Systematic Reviews and Meta-Analysis (PRISMA; Liberati et al. 2009) guidelines. The year for the study was selected from 1970 till now. Initially, 322 papers were identified by the Web of Science with the keywords. Only 215 were selected after removing duplicate records and all other areas excluding business, management, environmental studies, business finance, economics and ethics-related papers. After that, only article and review papers are chosen, leaving 212 papers in total. Out of these 212 papers, however, there were a few non-aligned papers. There were papers related to CSR policy, payment choice of M&A, analysts’ view on the acquisition, knowledge acquisition, effect of external societal norms on M&A, CEO turnover and CSR, socially responsible investment and firm value, etc. Consequently, they had to be removed for consideration as the underlying theme is the ESG effect on M&A. This eventually led to the selection of 41 papers. Then the same process was applied for Scopus and the search got zeroed down to 52 papers, out of which the 41 papers found in Web of Science were also present. Eventually, the common 41 papers from both were selected. The day the data were searched last was 24th of September, 2023 ( Figure 2).

### 4.3 Assessing

The final corpus is assessed through bibliometric analysis which uses quantitative methods to derive scientific information on scholarly researches ( Gepp et al., 2018). This approach proves advantageous in mitigating biases associated with manual and qualitative reviews, given their inherent susceptibility to errors and subjectivity ( Burton et al., 2020).

The bibliometric analysis encompasses performance analysis and science mapping, facilitated by network analysis utilizing keywords co-occurrence. This intricate process is executed utilizing Vos viewer, a tool recognized for its efficacy in visualizing and interpreting bibliometric data ( Leal Filho et al., 2018).

The study also examines potential areas for future research.By adopting rigorous bibliometric techniques, this research enhances the objectivity and robustness of the analysis, contributing valuable insights to the scholarly discourse in the field.

Finally, information bias is minimized by the investigators (authors of this article) by choosing the most relevant database and publications and removing the duplicate data. The first author was engaged in conceptualization, methodology, data curation, interpretation, writing-original draft, writing-review and editing and project administration. The rest of the authors were engaged in supervision, formal analysis, resources, interpretation, and editing. This ensured removal of any biases whatsoever.

## 5. Findings

The findings of this research are presented in two distinct sections: performance analysis and science mapping.

### 5.1 Performance analysis

Performance analysis serves as a comprehensive evaluation of a specific research domain, employing bibliometric techniques to rigorously profile its characteristics ( Donthu et al., 2021). This examination involves discerning the publication trend, identifying top-contributing journal articles, authors, institutions, and countries. Additionally, it delves into methodologies and contexts ( Donthu et al., 2021).

**5.1.1 Publication Trend for Sustainability and M&A Research**

The figure in Section 5.1.1 illustrates the panel data of publication trend. Mean TC per Art, denoting the average total citations per article, is calculated by dividing the cumulative number of times all authors’ articles have been cited by other articles by the total number of articles written by them, further divided by the citable years. Mean TC per Art is presented as Mean TC per Art divided by citable years. Notably, the number of articles (N) exhibits a steady increase, particularly over the last five years. The data suggests that the inaugural article on the subject of sustainability’s impact on mergers was published in 2013 ( Deng et al., 2013).

**5.1.2 Key findings on influential articles in sustainability and M&A research**

Table 1 illustrates the notable articles in the field of sustainable finance and M&A research, gauged by citation impact. ( Deng et al., 2013) article stands out as the most cited, accumulating a total of 515 citations, averaging 46.82 citations per year. Following closely are articles authored by Bereskin et al. (2018) in the Journal of Financial and Quantitative Analysis, Journal of Corporate Finance, and Finance Research Letters, respectively.

**Table 1.** Most influential articles for sustainability and M&A research.

Author(s)	Articles title	Source title	Year	TC	TC/Y
Deng, X., Kang, J. K., & Low, B. S.	Corporate social responsibility and stakeholder value maximization: evidence from mergers	*Journal of Financial Economics*	2013	515	46.82
Bereskin, F., Byun, S. K., Officer, M. S., & Oh, J. M.	The effect of cultural similarity on mergers and acquisitions: evidence from corporate social responsibility	*Journal of Financial and Quantitative Analysis*	2018	49	8.17
Arouri, M., Gomes, M., & Pukthuanthong, K.	Corporate social responsibility and M&A uncertainty	*Journal of Corporate Finance*	2019	41	8.2
Gomes, M., & Marsat, S.	Does CSR impact premiums in M&A transactions?	*Finance Research Letters*	2018	38	6.33
Chen, E., & Gavious, I.	Does CSR have different value implications for different shareholders?	*Finance Research Letters*	2015	38	4.22
Gomes, M. (2019).	Does CSR influence M&A target choices?	*Finance Research Letters*	2019	23	4.6
Gul, F. A., Krishnamurti, C., Shams, S., & Chowdhury, H.	Corporate social responsibility, overconfident CEOs and empire building: agency and stakeholder theoretic perspectives	*Journal of Business Research*	2020	19	4.75
Krishnamurti, C., Shams, S., Pensiero, D., & Velayutham, E.	Socially responsible firms and mergers and acquisitions performance: Australian evidence	*Pacific-Basin Finance of Journal*	2019	17	3.4
Tampakoudis, I., & Anagnostopoulou, E.	The effect of mergers and acquisitions on environmental, social and governance performance and market value: evidence from EU acquirers	*Business Strategy and The Environment*	2020	16	4
Yen, T. Y., & André, P.	Market reaction to the effect of corporate social responsibility on mergers and acquisitions: evidence on emerging markets	*Quarterly Review of Economics and Finance*	2019	15	3

TC Total citations, C/Y Average citations per year.

( Bereskin et al., 2018)’s work has garnered 49 citations, averaging 8.17 citations per year, while Arouri et al.’s research has been cited 41 times that boils down to 8.2 citations annually ( Arouri et al., 2019). Gomes and Marsat (2018) and Gomes (2019) have their researches published in Finance Research Letters receiving 38 and 23 citations, scoring 6.33 and 4.6 citations annually. 

Table 1 portrays the most influential articles in the area of sustainability and M&A research, gauged by citation impact. ( Deng et al., 2013) has been mentioned a total of 515 times since 2013 with an average of 46.82 articles per year. Deng followed closely ( Bereskin et al., 2018) and Arouri et al’s. (2019), whose works have been cited 49 and 41 with an average of 8.17 and 8.2 per year respectively.

**5.1.3 Notable journals for sustainability and M&A research**

Top contributing journals can be discussed in terms of most relevant journals, most cited journals, source impact, i.e., journal categorized by H-Index and source dynamics, i.e. growth of the paper publications of the most influential journals.

Figure 4 represents the most relevant sources graphically. Most relevant sources are the journals from which the papers have been identified. In other words, they are the most productive ones for sustainable financeand M&A research. Figure 5, on the other hand, represents journals whose references are quoted in the selected papers.

**Figure 3.**
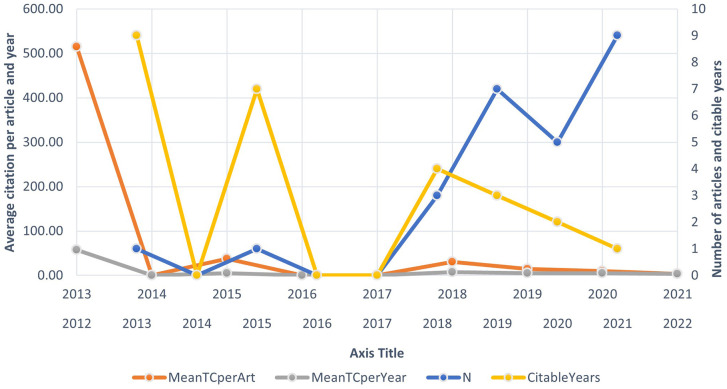
Yearly research growth.

**Figure 4.**
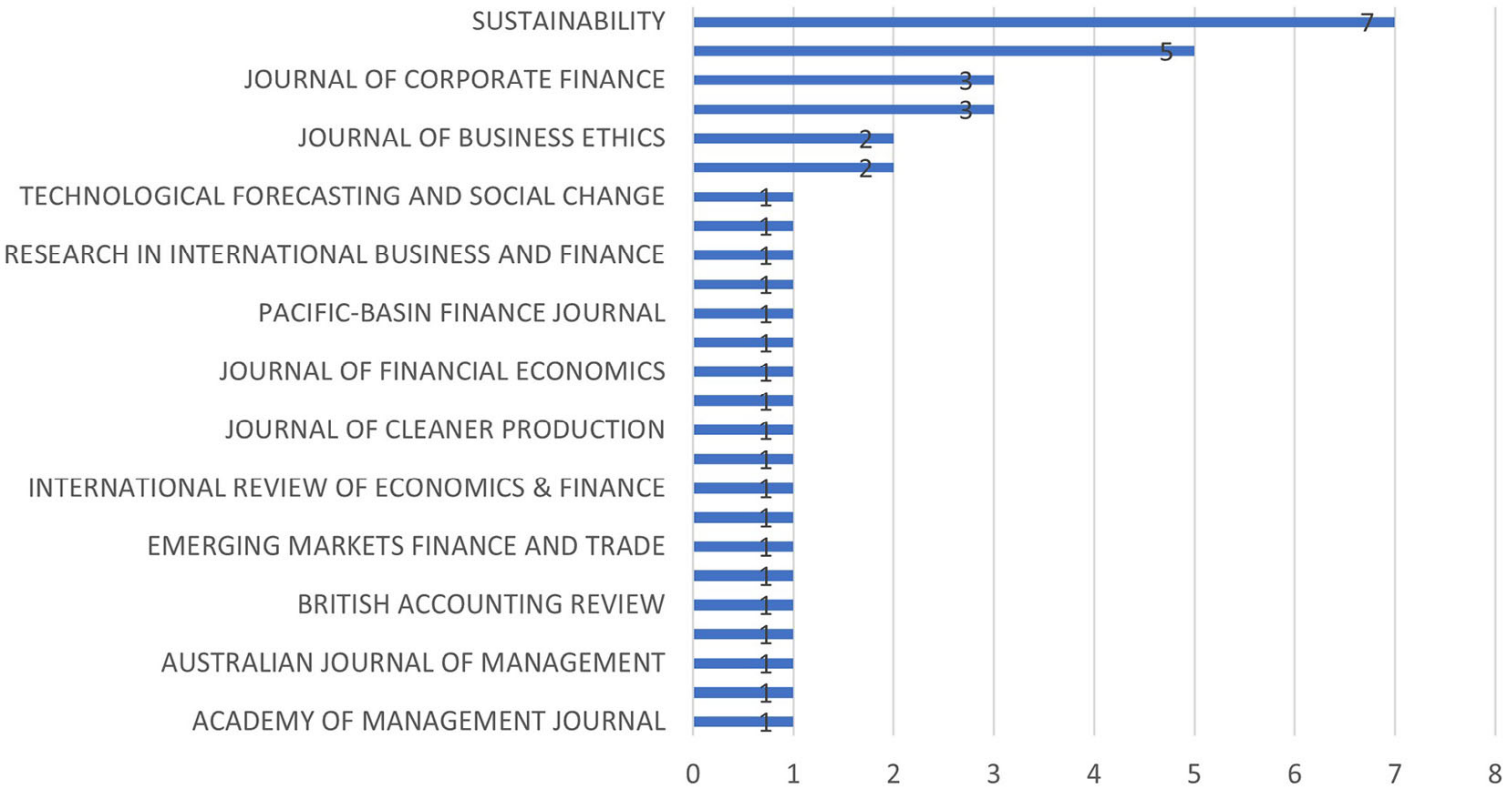
Most relevant sources.

**Figure 5.**
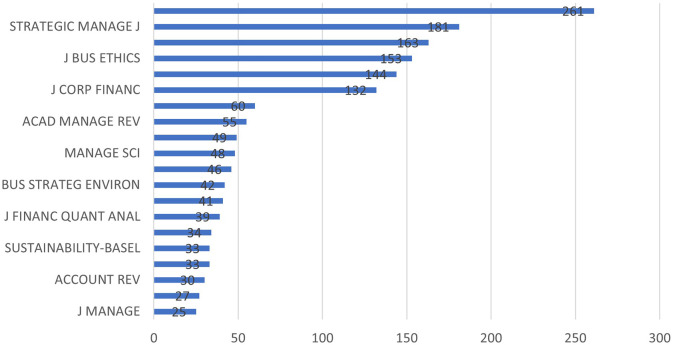
Most cited sources.

**Figure 6.**
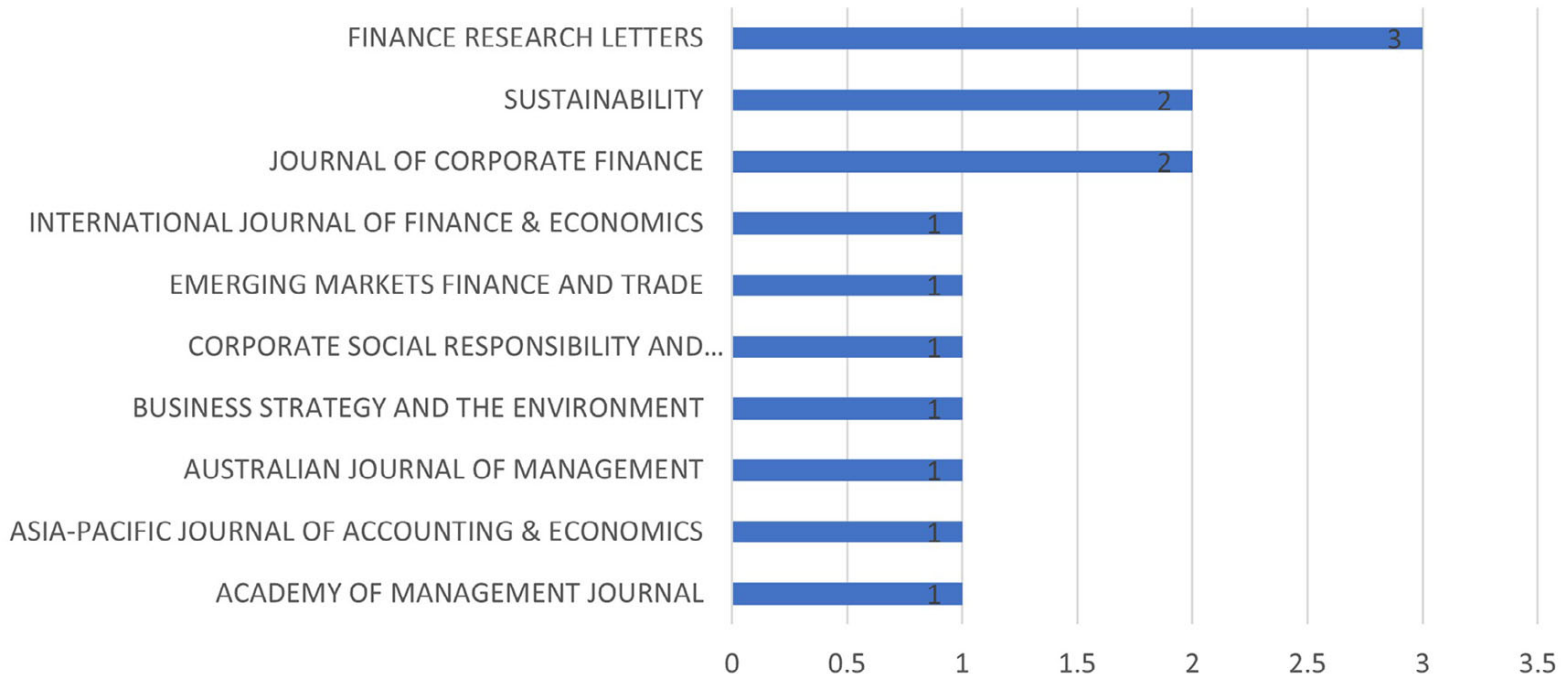
Source Impact (H-Index).

Figure 6 demonstrates the journals which has authors having most academic impact using H-Index as a measure. 

Table 2 is a testimony of the fact that top contributing journals may not have the largest submissions. Sustainability and Finance Research Letters are the largest contributors in sustainability and M&A research. However, Journal of Corporate Finance and Journal of Business Ethics have larger citations even though they have lesser publications than the earlier two mentioned.

**Table 2.** Top contributing journals for sustainability and M&A research (minimum 2 publications).

Journal	TP	TC	h
Sustainability	7	33	2
Finance Research Letters	5	34	3
International Review of Financial Analysis	3	7	1
Journal of Corporate Finance	3	132	2
Corporate Social Responsibility and Environmental Management	2	24	1
Journal of Business Ethics	2	153	1
Academy of Management Journal	1	60	1

TP Total publication, TC Total citation, h h-index.

**Figure 7.**
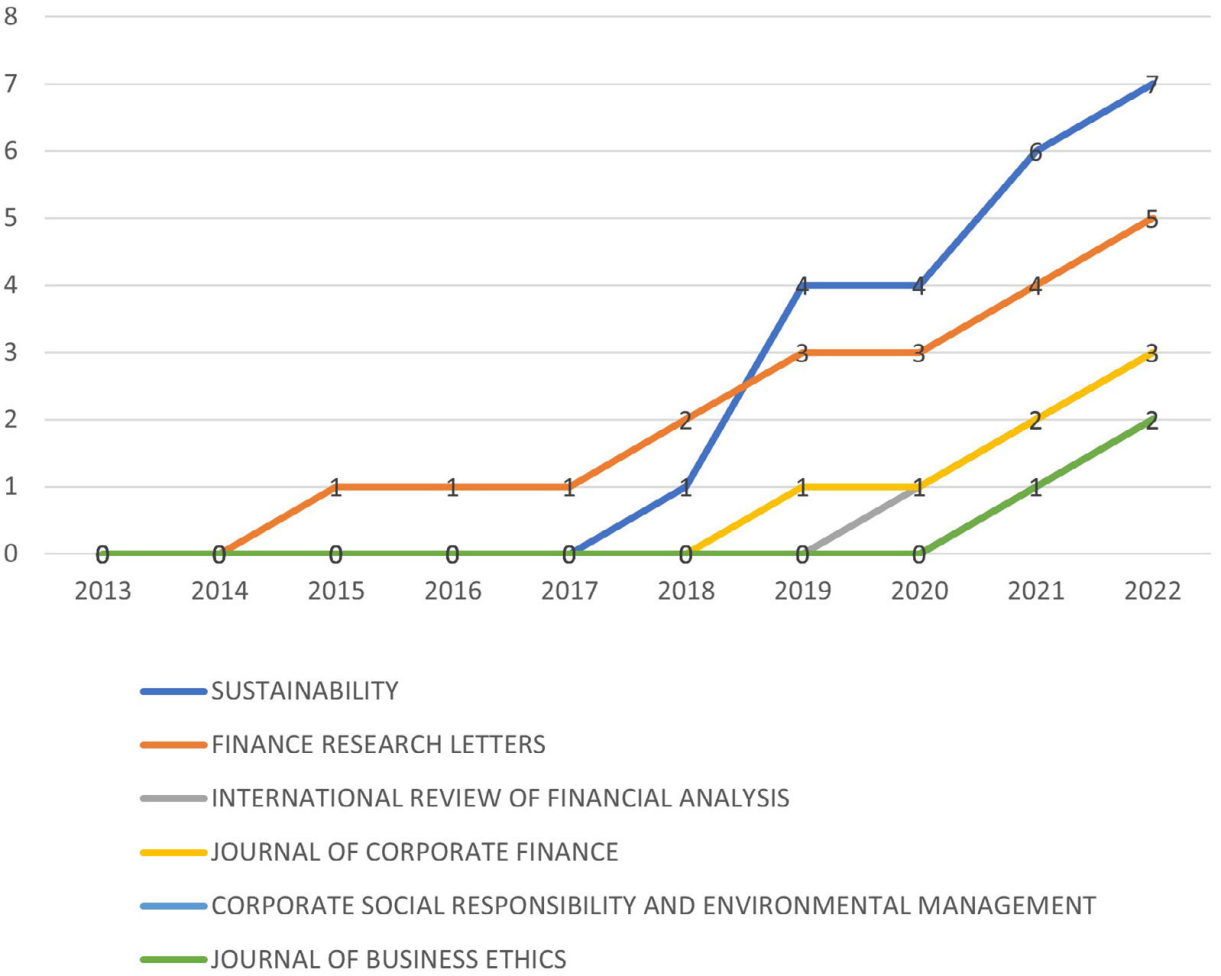
Source dynamics.

Figure 7 shows the growth of the number of papers on this area published in top ranked journals. As it can be observed that ‘Sustainability’ and ‘Finance Research Letters’ consists the maximum publication on this topic. It can also be observed that the growth starts from 2018 onwards.

**5.1.4 Notable authors for sustainability and M&A research**

Top contributing authors are presented in Table 3. Syed Shams from University of Southern Queensland, Australia and Mathiew Gomes from Université Clermont Auvergne, France, both of whom have contributed four and three each in the field of sustainability and M&A research. Among the top contributing authors, Mathiew Gomes is leading the chart who has 102 citations for his 3 papers.

**Table 3.** Notable authors for sustainability and M&A research (minimum 2 publications).

Author	Affiliation and country	TP	TCP		TC	TC/TP	TC/TCP	*h*
Shams S.	University of Southern Queensland, Australia	4	3		40	10.00	13.33	3
Gomes M.	Université Clermont Auvergne, France	3	3		102	34.00	34.00	3
Krishnamurti C.	University of South Australia, Australia	3	3		40	13.33	13.33	3
Tampakoudis I.	University of Macedonia, Greece	3	3		20	6.67	6.67	2
Chowdhury H.	The University of Queensland, Australia	2	2		23	11.50	11.50	2
Kiosses N.	University of Macedonia, Greece	2	2		4	2.00	2.00	1
Li Mh	East China Normal University, China	2	2		4	2.00	2.00	2
Liu M	Zhongnan University of Economics and Law, China	2	1		3	1.50	3.00	1
LU Wj	Wuhan University, China	2	1		3	1.50	3.00	1
Zhang F.	Tsinghua University, China	2	2		4	2.00	2.00	2

TP Total publication, TCP Total cited publication, TC Total citations, TC/TP Average citations per publication, TC/TCP Average citations per cited publication, h h-index; curated by the authors from the data collected from Biblioshiny

**5.1.5 Notable institutions for sustainability and M&A research**

As shown in Table 4, University of Macedonia, Greece and University of Southern Queensland, Australia has the maximum number of participations with 5 and 4 papers each. However, Université Clermont Auvergne, France has the maximum citation of 102 among all the institutes. The university also has highest average citations per cited publication.

**Table 4.** Notable institutions for sustainability and M&A research.

Institution	TP	TCP	TC	TC/TP	TC/TCP	h
University of Macedonia, Greece	5	5	24	4.80	4.80	2
University of Southern Queensland, Australia	4	3	40	10.00	13.33	3
Université Clermont Auvergne, France	3	3	102	34.00	34.00	3
University of South Australia, Australia	3	3	40	13.33	13.33	3
The University of Queensland, Australia	2	2	23	11.50	11.50	2
East China Normal University, China	2	2	4	2.00	2.00	2
Zhongnan University of Economics and Law, China	2	1	3	1.50	3.00	1
Wuhan University, China	2	1	3	1.50	3.00	1
Tsinghua University, China	2	2	4	2.00	2.00	2

TP Total publication, TCP Total cited publication, TC Total citations, TC/TP Average citations per publication, TC/TCP Average citations per cited publication, h h-index; curated by the authors from the data collected from Biblioshiny

**Table 5.** Top contributing countries for sustainability and M&A research.

Country	TP	TCP	TC	TC/TP	TC/TCP	h
Australia	9	8	103	11.44	12.88	8
China	8	6	14	1.75	2.33	6
Greece	5	5	24	4.80	4.80	2
France	3	3	102	34.00	34.00	3

TP Total publication, TCP Total cited publication, TC Total citations, TC/TP Average citations per publication, TC/TCP Average citations per cited publication, h h-index; curated by the authors from the data collected from Biblioshiny

**5.1.6 Top contributing countries for sustainability and M&A research**

France, China, Greece and France have the highest number of publications among all and Australia is leading the chart. Australia and France have the highest number of citations with 103 and 102 each. However, France yields the highest average citation of 34.

**5.1.7 Methodological choices and research contexts for sustainability and M&A research**

The research findings reveal a predominance of quantitative methodologies across all examined papers, with one exception providing a managerial framework for CSR in the context of cross-border M&A ( Parker et al., 2012). The majority of papers utilized regression models to test their hypotheses, employing M&A-related variables such as the number of M&A and M&A Ratio as dependent variables, while incorporating Corporate Social Responsibility (CSR) – measured by adjusted CSR – as an independent variable. Control variables encompassed Book leverage, cash, Ln of market cap, Tobin’s Q, ROA, Sales growth, Board size, CEO duality, and Fraction of independent directors.

The observed negative relationship between M&A deals and CSR scores aligns with stakeholder theory, suggesting that corporations with a stronger CSR orientation exhibit fewer M&A activities. Notably, only 12 out of 41 papers focused on multinational data and cross-border M&A, with just one addressing emerging markets. Furthermore, a substantial portion of the analyses predominantly relies on data from the USA and China. This underscores a potential disparity in the global commitment to sustainable practices, indicating a heightened dedication in the USA, China, and select European countries in contrast to the rest of the world.

### 5.2 Science mapping

**5.2.1 Temporal analysis using word clouds for sustainability and M&A research**

**Figure 8.**
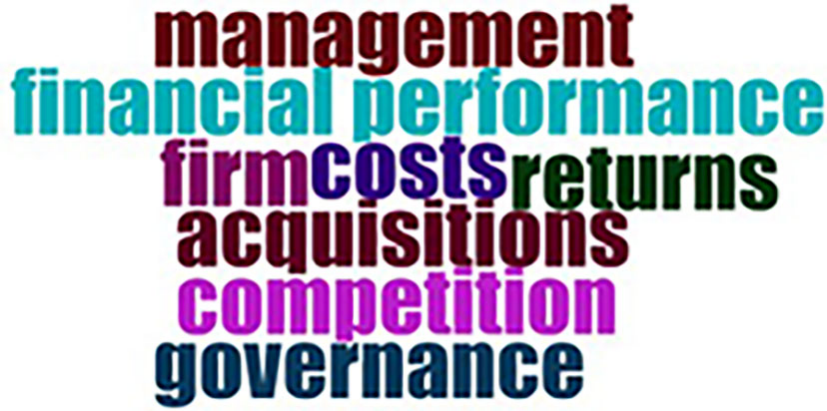
Sustainable finance and M&A research between 2010 and 2015.

Within the selected research period spanning from 1970 to 2022, it is noteworthy that the inaugural paper specifically addressing sustainability and M&A research emerged as recently as 2013. The body of literature on sustainability and M&A research is delineated into two distinct temporal segments: 2013 to 2017 and 2018-2022. A discernible pattern emerges in the initial five years, wherein key thematic focal points such as “management,” “financial performance,” “firms,” “costs,” “returns,” “acquisitions,” “competition,” and “governance” gained prominence, as exemplified in Figure 8 (E. Chen & Gavious, 2015; Cheng et al., 2014.; Deng et al., 2013).

**Figure 9.**
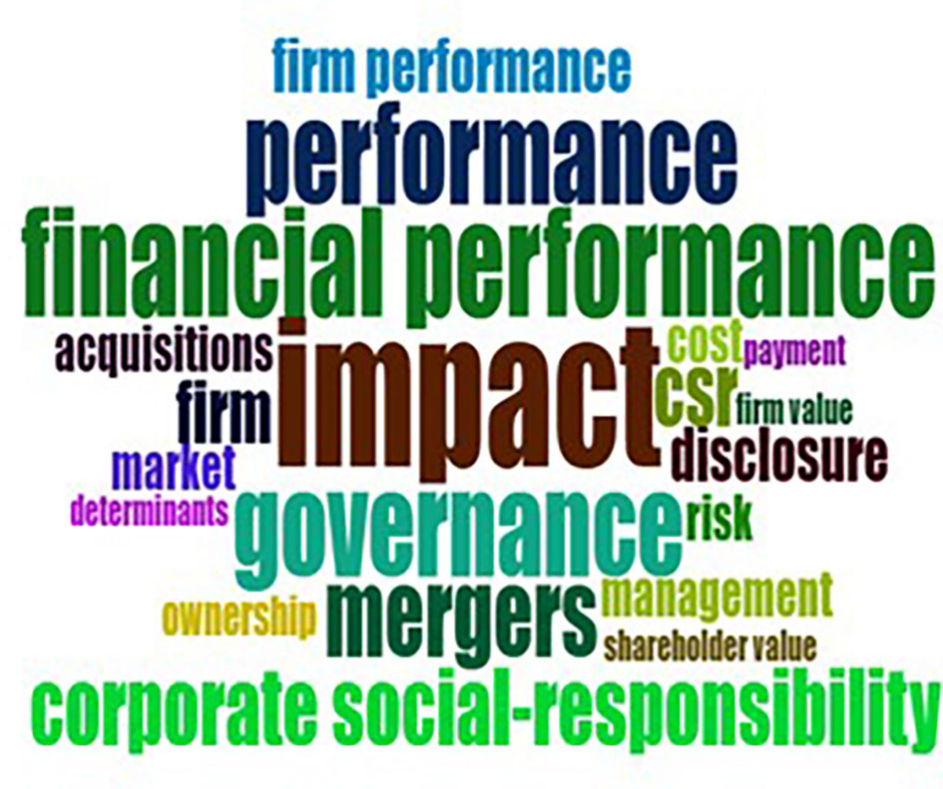
Sustainable finance and M&A research between 2016 and 2022.

Figure 9, on the other hand, has shown advent of new ideas including “impact”, “payment”, “market”, “determinants”, “ownership”, “corporate social responsibility”, “shareholder value”, “CSR”, “risk” and “firm value” among others ( Arouri et al., 2019; Obergassel et al., 2018).

**5.2.2 Network analysis**

In lieu of employing word clouds for temporal analysis, this study adopts network analysis, leveraging keyword co-occurrence within articles to unravel the prominent themes shaping the intellectual landscape of sustainability and M&A research. The chosen analytical approach is ‘co-occurrence,’ with the unit of analysis being ‘author keyword.’ Out of an initial pool of 103 keywords, only 16 meet the established threshold of a minimum of 2 occurrences.

**Figure 10.**
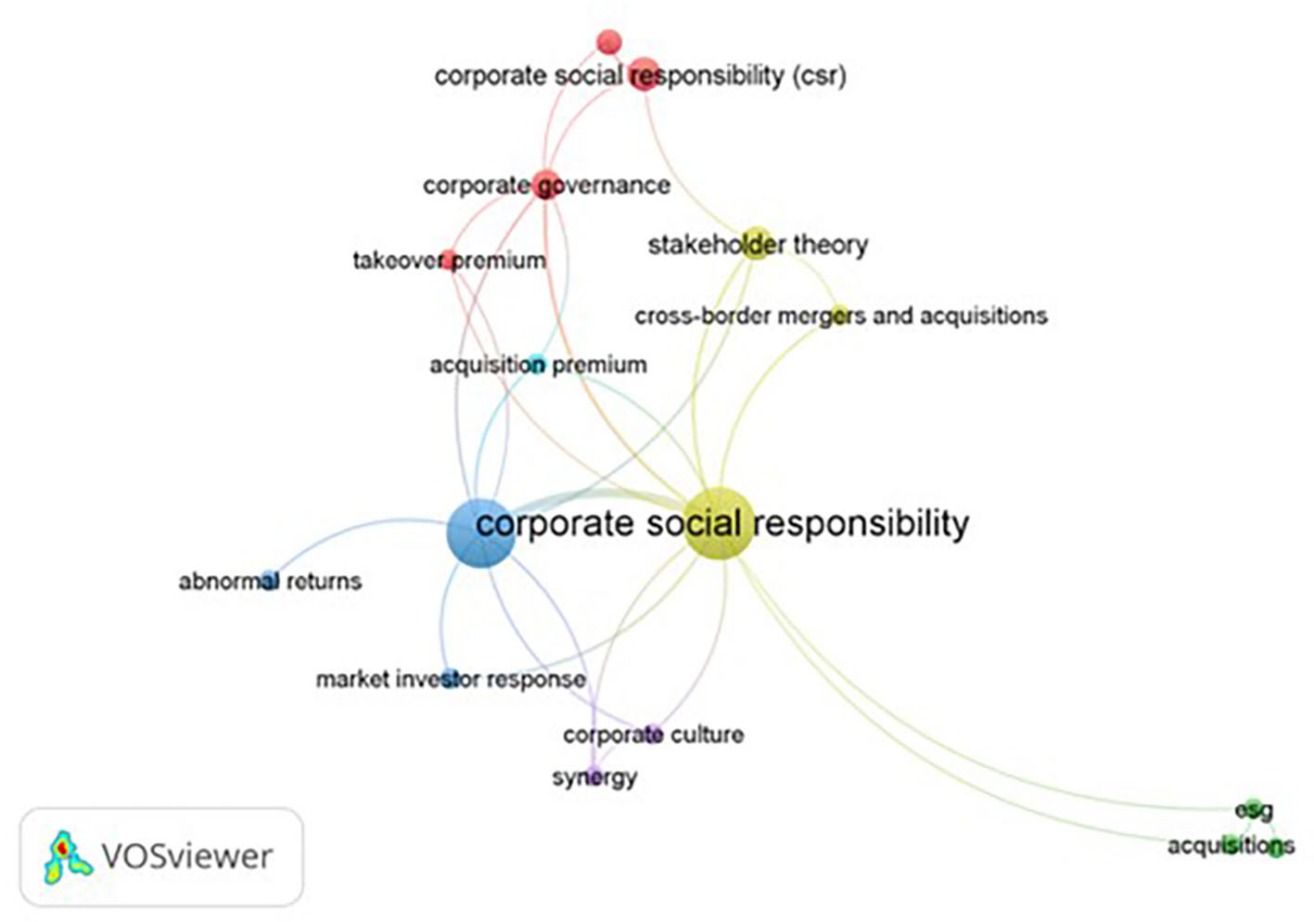
Keyword network of sustainability and M&A research.

Subsequently, clusters are formed based on the identified co-occurrences, as illustrated in Figure 10 and detailed in Table 6. Among the six clusters identified, Cluster 1 (depicted in red) emerges with the highest number of co-occurrences in author’s keywords, constituting 60.76% of the total occurrences. Cluster 4 (olive) follows, encompassing 15.19% of the total occurrences, while Cluster 3 represents 8.86% of the total occurrences. Notably, the concept of acquisition premium exhibits the least prevalence, accounting for a mere 1.53% of the total clusters.

**Table 6.** Major clusters and keywords for sustainability and M&A research.

Cluster	Keyword	Occurrences	Occurrences per cluster	Percentage of occurrence
Cluster 1 (Red)	Corporate social responsibility	22	48	60.76%
	M&A	20		
	Corporate governance	4		
	takeover premium	2		
Cluster 2 (Green)	Acquisitions	2	6	7.59%
	ESG	2		
	sustainability	2		
Cluster 3 (Deep blue)	abnormal returns	2	7	8.86%
	Market investor response	2		
	M&A	3		
Cluster 4 (Olive)	CSR	5	12	15.19%
	Cross border M&A	2		
	Stakeholder's theory	5		
Cluster 5 (Purple)	Corporate culture	2	4	5.06%
	Synergy	2		
Cluster 6 (Light Blue)	Acquisition premium	2	2	2.53%

This analysis reveals a discernible gap in research focus, particularly concerning corporate culture, synergy, and acquisition premium, suggesting that these areas remain relatively underexplored. The findings thus suggest potential avenues for future research, emphasizing the need for scholars to delve into these underdeveloped domains within the context of sustainability and M&A studies.

The summary of each of the clusters are presented below.

The table you provided summarizes the major clusters and keywords for sustainability and M&A (Mergers and Acquisitions) research. Here’s a brief explanation of each cluster:

Cluster 1 (Red): This cluster is primarily focused on “Corporate Social Responsibility” and “M&A”. It has the highest occurrence rate among all clusters, with “Corporate Social Responsibility” appearing 22 times and “M&A” appearing 20 times.

Cluster 2 (Green): This cluster is centered around “Acquisitions”, “ESG (Environmental, Social, and Governance)”, and “Sustainability”. Each keyword appears twice in this cluster.

Cluster 3 (Deep Blue): This cluster includes keywords such as “Abnormal Returns”, “Market Investor Response”, and “M&A”. “Abnormal Returns” and “Market Investor Response” each appear twice, while “M&A” appears three times.

Cluster 4 (Olive): This cluster is focused on “CSR (Corporate Social Responsibility)”, “Cross Border M&A”, and “Stakeholder’s Theory”. “CSR” appears five times, while “Cross Border M&A” and “Stakeholder’s Theory” each appear twice.

Cluster 5 (Purple): This cluster includes “Corporate Culture” and “Synergy”, each appearing twice.

Cluster 6 (Light Blue): This cluster is solely focused on “Acquisition Premium”, which appears twice.

These clusters were curated by the authors from the data collected from Biblioshiny, a tool for bibliometric analysis. Each cluster represents a different aspect or theme within the broader field of sustainability and M&A research.

## 6. Linking up the past, present and the way forward

Diverse Theoretical Frameworks: The literature review reveals that research on the impact of CSR/ESG activities on mergers and acquisitions is grounded in various theoretical frameworks. Stakeholder theory, resource-based theory, agency theory, and conflict resolution theory are identified as the primary lenses through which scholars have examined the positive or negative effects of CSR/ESG activities on acquisition outcomes ( Barros et al., 2022; Kristian et al., 2021; Singh et al., 2023).

Stakeholder Theory Perspective: Stakeholder theory propagates that engaging in socially responsible actions can enhance a firm’s reputation, leading to positive impacts on customer loyalty, trust, and identification. This, in turn, contributes to improved revenue and profit outlook, positively influencing share prices. CSR activities align stakeholders’ interests, reduce information asymmetry thereby enhancing investor confidence leading to improved efficiency in M&A deals and enhanced shareholder wealth ( Brower and Mahajan, 2013.; Freeman et al., 2004; Freudenreich et al., 2020).

SShareholder Theory Perspective: Conversely, shareholder theory critiques stakeholder theory by emphasizing the potential flaw of ignoring the cost of CSR investments. It reasons that the main goal of a business is to boost asset utilization and that consistent CSR activities may divert resources from productive activities. Previous research has demonstrated an inverse relationship between CSR/ESG activities and announcement returns, highlighting a conflicting perspective on the effect of CSR in M&A( How et al., 2019; Tse, 2011).

Mixed Evidence on CSR Impact: The literature review highlights conflicting evidence regarding the effect of CSR on M&A announcements, with both positive and negative effects supported by various researchers. As such, there is a need to explore the association between the ESG performance of acquirers and announcement returns in domestic M&A deals( Deng et al., 2013).

Evolution of Market Reaction: The study proposes that, initially, CSR/ESG activities may put a firm at a competitive disadvantage, diminishing shareholder wealth. However, over time, as market reputation and goodwill increase, the adverse market reaction may transform into a favorable market return, forming a U-shaped pattern ( Bose, 2020).

ESG Metrics and Reporting: TThe frequent use of ESG metrics has changed the way of CSR activities reporting. A large number of researchers have started using ESG, as a proxy for corporate sustainability and this eventually have started playing a crucial role in evaluating corporate performance post-acquisition.Though economic parameters are often used in the context of ESG, there is a gap in research focusing on announcement return and market value in the context of M&A ( Kristian et al., 2021; Singh et al., 2023).

Governance and CSR Cultures: Governance performance and CSR practices of acquirer firms are identified as crucial factors influencing announcement returns in M&A. Higher governance performance coupled with compatibility in CSR cultures are linked with higher acquisition returns ( Lu and Wang, 2021; Thanetsunthorn and Wuthisatian, 2016).

PPositive Association Between CSR and Market Value: Contemporary research suggests a positive relationship between CSR and market value, reflected by metrics like Tobin’s Q. Acquirers with superior ESG performance are found to experience significant effects on their market value, emphasizing the need of considering CSR in assessing expectations from market, financials, and reputational effects ( Bajic & Yurtoglu, 2018; Lee, 2020).

Future Research Agendas: This review emphasizes the need for further research on the relationship between CSR/ESG activities and M&A outcomes, particularly in the areas of announcement return and market value. The paper sets the agenda for future investigations by proposing research agenda after summarizing key findings from the last decade ( Li et al., 2023; Singh et al., 2023).

The research outcomes can be categorized systematically into four distinct areas, each providing valuable insights into the dynamics of CSR and ESG considerations within the context of M&A

Comparative Analysis of Bidder CSR and Target CSR:

The first stratum involves a comprehensive examination of the CSR practices of both acquirer and target entities. The findings illuminate the existing disparities and similarities in CSR commitments, shedding light on the alignment or misalignment of social responsibility values between merging entities ( Salvi et al., 2018).

Impact of Bidder’s CSR/ESG Performance on M&A Outcomes:

This stratum delves into the nuanced relationship between the bidder’s CSR and ESG performance and the outcomes of M&A transactions. Through a meticulous analysis, the research uncovers the influence of corporate social and environmental practices on the overall success and implications of mergers and acquisitions.

Effects of M&A Transactions on CSR Practices:

Examining the reciprocal relationship, this stratum investigates how M&A activities impact the CSR initiatives of the involved entities. The findings highlight the transformative effects of M&A on CSR frameworks, providing valuable insights into the evolving nature of CSR commitments within the ambit of organizational consolidation ( Qiao et al., 2018).

Impact of Target’s CSR/ESG Performance on M&A Dynamics:

This stratum focuses on the target entities’ CSR and ESG performance and its consequential influence on the dynamics of mergers and acquisitions. Through rigorous analysis, the research discerns the role of target corporations’ social and environmental practices in shaping the overall trajectory and success of M&A transactions ( Qiao et al., 2018).

These stratified findings contribute a detailed roadmap of the multifaceted interactions between CSR, ESG considerations, and M&A, offering valuable insights for practitioners, policymakers, and scholars alike in navigating the complex landscape of sustainable business practices within the realm of mergers and acquisitions.

### 6.1 CSR performance comparison between acquirer and target companies

**Findings**

The research in this domain is classified into four distinct categories on the basis of the research objectives. Papers in the first three categories examine whether there is a synergistic gain when the bidder firm’s Corporate Social Responsibility (CSR) practices surpass, equal, or fall below those of the target firm (Category 1, 2, and 3). The fourth category explores synergistic gains in cases where there is a difference in CSR practices between the bidder and target firms. These tests are conducted with a focus on either acquisition synergy, representing the positive movement in the acquirer’s share price due to the M&A, or deal premium, the premium paid by the acquiring firm.

The research outcomes indicate that a superior governance performance of the acquirer firm compared to the target firm is associated with higher synergistic gains ( Cheng et al., 2014). Additionally, socially irresponsible behavior exhibited by a target firm correlates with a reduction in the acquisition or deal premium offered by the acquirer (C. Chen et al., 2022). Another noteworthy finding is that a significant difference in CSR culture between the acquirer and target can diminish the acquirer’s announcement and long-term returns ( Alexandridis et al., 2022). These key findings are summarized in Table 7.

**Table 7.** Literature on comparison between bidder CSR and target CSR.

Category	acquisition synergy/deal premium	Authors' Names	Title	Sample	Country	Findings
Bidder CSR > Target CSR	Acquisition synergy	Chen, C., Lu, W., & Liu, M.	corporate social responsibility learning in mergers and acquisitions	396 M&A deals	USA (1990-2004)	acquirer firm governance>target firm governance, there is higher synergetic gain.
		Hussain, T., & Shams, S.	pre-deal differences in corporate social responsibility and acquisition performance	1334 M&A deals	International (2003-2016)	Higher the acquirer's synergy score compared to the bidder, higher the combined CAR.
	Deal premium	Maung, M., Wilson, C., & Yu, W.	does reputation risk matter? evidence from cross -border mergers and acquisitions	248 M&A deals	International (2007-2017)	Socially irresponsible behaviour of a target firm leads to less acquisition premium or deal premium by the acquirers. If the target's csr< acquirer's csr, there are low acquisition premium.
Bidder CSR < Target CSR	Deal premium	Cho, K., Han, S. H., Kim, H. J., & Kim, S	the valuation effects of corporate social responsibility on mergers and acquisitions: evidence from target firms	199 M&A deals	USA (1993-2016)	target CSR performance > acquirer CSR performance, higher premium for target shareholders.
		Li, K., He, C., Dbouk, W., & Zhao, K.	the value of csr in acquisitions: evidence from china	2224 M&A deals	China (2007-2018)	If targets have higher CSR performance than the acquirer, then deal premium is significant or acquisition valuation is high.
Bidder CSR = Target CSR	Acquisition synergy	Bereskin, F., Byun, S. K., Officer, M. S., & Oh, J. M.	the effect of cultural similarity on mergers and acquisitions: evidence from corporate social responsibility	570 M&A deals	USA (1994-2014)	Firms with similar CSR culture augments higher merger returns.
		Doukas, J. A., & Zhang, R.	managerial ability, corporate social culture, and m&as	18661 M&A deals	USA (1992-2017)	If the acquiring firm has same similar CSR practice as the target firm, post-merger returns improve.
Generic	Acquisition synergy	Alexandridis, G., Hoepner, A. G., Huang, Z., & Oikonomou, I.	corporate social responsibility culture and international m&as	220 M&A deals	Developed market (2004-2012)	Difference in CSR culture between acquirer and target can lower the acquirer announcement and long-term return.

It is noteworthy that existing scholarly discourse does not extensively cover the application of novel sustainable financing tools, such as green bonds, and their role in value generation in the context of Mergers and Acquisitions (M&A) ( Salvi et al., 2018). The case of China, where governmental actions in conjunction with market mechanisms have stimulated investments promoting environmental sustainability, serves as a significant illustration (X. Chen et al., 2023). Consequently, forthcoming research could probe into the utilization of such novel financing instruments and their implications in M&A operations.

**Future Research Directions**

Does funding sustainable investments through innovations like green bonds improve acquisition synergy?

What is the optimal funding approach for sustainable finance by acquiring firms to achieve above-average synergistic gains in M&A transactions?

How can acquiring firms optimize funding through innovative sustainable financial instruments to achieve above-average synergistic gains in M&A transactions?

### 6.2 Effect of bidder’s CSR/ESG performance on M&A

Existing research in this domain has primarily focused on assessing the positive and negative impacts of aExisting research in this domain has primarily focused on assessing the positive and negative impacts of acquisitions stemming from the acquiring firm’s CSR performance. Similar to the aforementioned category, these evaluations predominantly consider acquisition synergy, denoting the positive movement in the acquirer’s share price resulting from the merger and acquisition (M&A) process, or the deal premium, which represents the amount paid by the acquiring firm. Table 8 provides a concise summary of the findings from these studies.

**Table 8.** Literature on effect of bidder's CSR/ESG performance on M&A.

Category	acquisition synergy/deal premium	Authors' Names	Title	Sample	Country	Findings
Positive effect	Acquisition synergy	Zhang, F., Li, M., & Zhang, M.	Chinese financial market investors attitudes toward corporate social responsibility: evidence from mergers and acquisitions	3000 M&A deals	China (2010-2017)	High CSR scores lead to high market return.
		Deng, X., Kang, J. K., & Low, B. S.	corporate social responsibility and stakeholder value maximization: evidence from mergers	1556 M&A deals	USA (1992-2007)	Acquirer with high CSR gets high CAR on announcement.
		Gomes, M.	does CSR influence M& A target choices?	608 M&A deals	International (2003-2014)	CSR performance improves merger value for both acquirers and targets.
		Mihaiu, D. M., Șerban, R. A., Opreana, A., Țichindelean, M., Brătian, V., & Barbu, L.	the impact of mergers and acquisitions and sustainability on company performance in the pharmaceutical sector	492 M&A deals	International (2010-2020)	High ESG score lead to high post-merger performance.
		Zhang, T., Zhang, Z., & Yang, J.	when does corporate social responsibility backfire in acquisitions? signal incongruence and acquirer returns	493 M&A deals	International (2002-2012)	Better CSR performance lead to better acquisition performance barring hostile takeovers.
		Krishnamurti, C., Shams, S., Pensiero, D., & Velayutham, E.	socially responsible firms and mergers and acquisitions performance: australian evidence	776 M&A deals	Australia (2000-2016)	Announcement day CAR is positive and significant when CSR-firm bids for acquisition. Also, CSR firms pay lower bid premium to target firms.
		Kim, B. J., Jung, J. Y., & Cho, S. W.	can esg mitigate the diversification discount in cross-border m&a?	129 M&A deals	Emerging market (2012-2018)	ESG can
						serve as a strategy for boosting business efficiency in cross-border M&A.
		Gul, F. A., Krishnamurti, C., Shams, S., & Chowdhury, H.	corporate social responsibility, overconfident ceos and empire building: agency and stakeholder theoretic perspectives	16635 M&A deals	USA (1996-2015)	Impact of CSR in M&A is contingent of CEO overconfidence. CSR increases the value of acquisition if CSEO is less overconfident and vice versa.
		Guidi, M., Sogiakas, V., Vagenas-Nanos, E., & Verwijmeren, P.	spreading the sin: an empirical assessment from corporate takeovers	23786 M&A deals	Intenational (1985-2015)	Shareholder's of acquirer discount sin acquisition.
		Hussaini, M., Hussain, N., Nguyen, D. K., & Rigoni, U.	is corporate social responsibility an agency problem? an empirical note from takeovers	564 M&A deals	USA (1992-2014)	Higher CSR performance of acquirer leads to higher takeover premium.
		Chen, E., & Gavious, I.	does CSR have different value implications for different shareholders?	134 M&A deals	Israel (2007-2012)	Positive pricing in M&A happens if acquirer is engaged in CSR used for mankind and not for the ones which is benefecial to the firm only.
		Petridis, K., Tampakoudis, I., Drogalas, G., & Kiosses, N.	a support vector machine model for classification of efficiency: an application to M&A	441 M&A deals	EU (2003-2017)	Performance of M&A deals is positively affected by gender diversity.
		Caiazza, S., Galloppo, G., & Paimanova, V.	the role of sustainability performance after merger and acquisition deals in short and-term	757 M&A deals	USA (2000-2019)	Companies with low or high ESG scores significantly impact the CAR value but the impact is weak. Also there is no significant difference between high and low CSR values CAR. Instead, long term performance is correlated to current CSR performance in the time of merger.
		Shi, J., Yu, C., & Li, Y.	beyond linear: the relationship between corporate social responsibility and market reactions to cross-border mergers and acquisitions	409 M&A deals	China (2010-2018)	not moderate, but low or high level of CSR affects market return.
		Fairhurst, D. D., & Greene, D. T.	too much of a good thing? corporate social responsibility and the takeover market	1596 M&A deals	USA (1996-2016)	Firms with high or low CSR scores experience a greater
						likelihood of takeover and lower wealth gains in takeovers relative to firms with moderate policies.
		Arouri, M., Gomes, M., & Pukthuanthong, K.	corporate social responsibility and m&a uncertainty	726 M&A deals	International (2004-2016)	High CSR will lead to lower uncertainty in terms of shareholder opposition, regulatory intervention, financing problems or internal target.
						resistance
		Yen, T. Y., & André, P.	market reaction to the effect of corporate social responsibility on mergers and acquisitions: evidence on emerging markets	1986 M&A deals	emerging markets (2008-2014)	Effect of CSR on M&A depends on cost benefit concern of investors. It's not CSR performance but agency cost (CSR related) which affects the market reaction.
		Qiao, M., Xu, S., & Wu, G.	corporate social responsibility and the long-term performance of mergers and acquisitions: do regions and related-party transactions matter?	1090 M&A deals	China (2012-2014)	buyers’ CSR performance
						has a significant and positive effect on long-term M&A performance.
Negative effect	Acquisition synergy	Wang, Z., Lu, W., & Liu, M.	corporate social responsibility overinvestment in mergers and acquisitions	614 M&A deals	USA (1996-2017)	If the acquiree is overinvested in CSR, the M&A deal value reduces. Also, financial performance post M&A declines.
		Krishnamurti, C., Shams, S., & Chowdhury, H	evidence on the trade-off between corporate social responsibility and mergers and acquisitions investment	8564 M&A deals	USA (1999-2016)	Negative relationship between M&A deals and CSR scores.
		Tampakoudis, I., Andrikopoulos, A., Nerantzidis, M., & Kiosses, N.	does boardroom gender diversity affect shareholder wealth? evidence from bank mergers and acquisitions	1130 M&A deals	USA (2003-2018)	Board gender diversity has a negative relationship with sdhafreholder wealth.
	Deal premium	Jost, S., Erben, S., Ottenstein, P., & Zülch, H.	does corporate social responsibility impact mergers & acquisition premia? new international evidence	1598 M&A deals	International (2003-2018)	neither acquirers’ nor targets’ CSR performance alone does significantly impact M&A premia.
No effect	Acquisition synergy	Li, M., Lan, F., & Zhang, F.	why chinese financial market investors do not care about corporate social responsibility: evidence from mergers and acquisitions	3500 M&A deals	China (2010-2017)	CSR doesn't affect investors' judgement on merger.

Majority of the publication analyzed indicate a positive association between high CSR performance and post-acquisition market returns ( Gomes, 2019). Additionally, certain studies highlight that long-term performance, as opposed to short-term acquisition gains, is associated with the current CSR performance at the time of merger ( Caiazza et al., 2021). It is noteworthy that shareholder theory posits that the cost of a CSR investment is disregarded, though no research has explored how the cost of CSR might impact M&A transactions.

Given these insights, the future research directions are articulated through the following research questions:

Is there an apparent connection between the sustainability performance (ESG) of the acquirer and the announcement return?

Does the association between sustainability performance (ESG) and the announcement return in domestic M&A transactions follow a linear or curvilinear trajectory, suggesting that ESG performance is inversely associated to announcement return until a certain point, beyond which it turns positive?

What are the quantifiable or measurable strengths, weaknesses, opportunities, or threats in relation to the sustainable investments of the acquirer, affecting the value of the new firm post-acquisition in both the short and long term?

How can the cost of sustainable investments by the acquirer be conceptually framed, considering various stakeholders, during an M&A transaction event?

### 6.3 Effect of M&A on CSR

The research in this domain adopts a novel approach, establishing sustainability (ESG) as a dependent variable and the propensity of M&A transactions as an independent variable in a reverse framework. Noteworthy findings across various studies assert that the post-merger ESG performance of the acquiring entity experiences enhancement subsequent to acquiring a target with superior ESG performance than that of the acquirer in the pre-merger stage. Moreover, a positive correlation is observed between the post-merger market value of the acquiring entity and its increased post-merger ESG performance relative to the pre-merger stage ( Tampakoudis et al., 2021).

Table 9 succinctly presents an overview of these seminal papers. The delineation of future research directions is encapsulated through the formulation of pertinent research questions:

**Table 9.** Literature on effect of M&A on CSR.

category	Authors' Names	Title	Sample	Country	Findings
Positive effect	Yang, N., Zhang, Y., Yu, L., Wang, J., & Liu, X.	cross-border mergers and acquisitions, regional cultural diversity and acquirers' corporate social responsibility: evidence from china listed companies	478 M&A deals	China (2007-2018)	Cross border M&A has a significant impact on acquirer's CSR.
	Tampakoudis, I., & Anagnostopoulou, E.	the effect of mergers and acquisitions on environmental, social and governance performance and market value: evidence from eu acquirers	100 M&A deals	Europe (2003-2017)	post-merger ESG performance of the acquirer increases following the acquisition of a target that has higher ESG performance than that of the acquirer in the premerger stage, whereas the post-merger market value of the acquirer increases following an increase in the acquirer's post-merger ESG performance in relation to its premerger ESG performance.
	Barros, V., Matos, P. V., Sarmento, J. M., & Vieira, P. R.	M&A activity as a driver for better ESG performance	14595 M&A deals	International (2002-2020)	M&A deals have positive impact on ESG scores.
	Hong, X., Lin, X., Fang, L., Gao, Y., & Li, R.	application of machine learning models for predictions on cross-border merger and acquisition decisions with ESG characteristics from an ecosystem and sustainable development perspective	85189 M&A deals	International (2009-2018)	M&A is a tool to improve sustainable development practices.

Which industries demonstrate notable improvements in sustainability performance in the short-term and long-term aftermath of M&A transactions?

In instances where the impact of M&A transactions on Corporate Social Responsibility (CSR) appears insignificant within specific industries, what factors contribute to this observed outcome?

What roles do regulators play, and what impacts do they exert, to ensure that M&A transactions effectuate a definitive enhancement in sustainable performance for the newly formed entity?

### 6.4 Effect of target’s CSR/ESG performance on M&A

Table 10 presents a comprehensive overview of the findings derived from the examined papers. Notably, the collective body of research underscores a consistent theme—namely, that the sustainable performance of the target company significantly influences both acquisition synergy and the associated deal premium.

**Table 10.** Literature on effect of target's CSR/ESG performance on M&A.

Category	Acquisition synergy/Deal premium	Authors' names	Title	Sample	Country	Findings
Positive effect	Acquisition synergy	Gomes, M., & Marsat, S.	does CSR impact premiums in M&A transactions?	588 M&A deals	International (2003-2014)	Target CSR is positively associated with M&A premium.
		Tong, L., Wang, H., & Xia, J.	stakeholder preservation or appropriation? the influence of target CSR on market reactions to acquisition announcements	237 M&A deals	USA (2000-2012)	Target CSR performance positively affects the CAR of acquirer in M&A announcement.
	Deal premium	Ozdemir, O., Binesh, F., & Erkmen, E.	the effect of target's CSR performance on M&A deal premiums: a case for service firms	277 M&A deals	USA (1996-2018)	target’s pre-acquisition
						CSR performance is positively related to deal premium. Ther premium is more in service industries than in non service industries.
		Qiao, L., & Wu, J.	pay for being responsible: the effect of target firm's corporate social responsibility on cross-border acquisition premiums	252 M&A deals	International (1991-2016)	Target firm's CSR affects cross border acquisition premium.

The identified research directions for future exploration are succinctly encapsulated in the following research questions:

To what degree does the alignment of the target company’s Environmental, Social, and Governance (ESG) performance with that of the acquirer contribute to the enhancement of acquisition synergy?

What observable changes in sustainable practices may be detected within the target company in the years preceding the M&A deal?

These research questions serve as pivotal inquiries guiding prospective investigations into the intricate dynamics of sustainable performance in the context of mergers and acquisitions.

Does funding sustainable investments through innovations like green bonds improve acquisition synergy?

What is the optimal funding approach for sustainable finance by acquiring firms to achieve above-average synergistic gains in M&A transactions?

How can acquiring firms optimize funding through innovative sustainable financial instruments to achieve above-average synergistic gains in M&A transactions?

## 7. Conclusion

In conclusion, this research employs a systematic literature review approach coupled with bibliometric analysis to offer valuable insights into the scholarly research on value creation through Mergers and Acquisitions (M&A) with a focus on sustainable practices. The utilization of machine learning and big data analytics contributes to a comprehensive understanding for researchers seeking to explore the historical and current role of sustainability in M&A.

The research underscores and brings to light the most impactful publications, scholarly articles, institutions, nations, and methodological preferences, concurrently revealing the chronological progression of research themes. This examination yields four primary insights and their corresponding implications.

Firstly, a consistent upward trend in publications from 2017 onwards, post the launch of the Sustainable Development Goals in 2016, underscores the growing significance of sustainability in M&A research. Notably, Australia and China lead in publications, and there is a call for more exploration in developing economies, particularly in the context of sustainable finance.

Secondly, the prevalence of quantitative research in this area is evident, yet the neglect of behavioral finance aspects in sustainable finance is apparent. The study advocates for a deeper exploration of the behavioral patterns of corporate representatives in sustainable investments, emphasizing the importance of understanding personality and behavioral biases.

Thirdly, the research points out the dominance of domestic data over multinational M&A data. Given the increasing trend in multinational M&As, future research should focus on multinational data and cross-border M&As to provide a more comprehensive understanding of the landscape.

Fourthly, the identification of new areas such as greenwashing and the use of artificial intelligence in sustainable finance highlights the need for future research exploration. Green audit emerges as a critical factor in verifying sustainable claims, while the incorporation of topics like artificial intelligence is essential for a holistic analysis of sustainable finance.

While acknowledging the effort to encompass relevant papers, it is crucial to note the limitation of relying on the Web of Science and Scopus databases. Moreover, However, this limitation does not compromise the enriching insights provided by the current analysis, offering a foundation for future researchers in this dynamic field of sustainable finance and M&A.

## Data Availability

Data for the article “Navigating the evolution: a systematic review of sustainable finance in mergers and acquisitions” is presented in https://doi.org/10.5281/zenodo.11185041 . The project title in repository is Sustainability_M&A. The repository contains following files namely prisma model and prisma checklist. The extended data is presented along with the prisma related files in the repository and is named as ESG export file. Data are available under the terms of the CC0 1.0 Universal. I have obtained permission from all authors to include their name and affiliation.
