# Supplementary material for: Navigating the evolution: a comprehensive review of sustainable finance in mergers and acquisitions
Source: F1000Res. 2025 Mar 14;14:298. [Version 1] doi: 10.12688/f1000research.150207.1 (PMC12041844; doi:10.12688/f1000research.150207.1)
Supplement: Peer review history

# Reviewer report: Reviewer response for version 1

**DOI:** 10.5256/f1000research.164752.r373245

**Published:** 2025-05-20

**Authors:** Dr. Asheetu Bhatia Sarin

**Affiliations:** 1 Guru Gobind Singh Indraprastha University, Dwarka, Delhi, India

The literature review should be comprehensive, and recent /up-to-date studies should be added. The interpretation of results is logical and related to the results.

 The conclusion should summarize findings and offer insights into managerial, practical, and future research implications.

 The author may use a professional editing service to improve readability.

**Navigating the evolution: a comprehensive review of sustainable finance in mergers and acquisitions**

**Mondal S, Rakshit D, Jee K and Tiwari SC**

**Peer Review Report**

 This paper offers a thorough and methodical review of sustainable finance within the context of mergers and acquisitions (M&A), shedding light on how environmental, social, and governance (ESG) considerations are increasingly shaping corporate deal-making. It distinguishes itself through a robust analytical approach, combining bibliometric techniques and machine learning to analyze 41 influential studies sourced from Web of Science and Scopus. The review traces evolving research patterns, particularly the surge in sustainability focused M&A since 2016, with China and Australia emerging as leading contributors.

 The study highlights key research gaps, including the limited focus on emerging markets, insufficient exploration of greenwashing risks, and the potential of artificial intelligence in M&A analysis. It offers a well-organized synthesis of theoretical frameworks such as stakeholder and shareholder theories alongside empirical findings that illustrate how ESG integration can improve M&A outcomes.

 By organizing the literature into clear categories such as comparisons of CSR between acquirers and targets, ESG's influence on deal success, and sustainability performance after mergers, the paper delivers clear and actionable insights. From a practical standpoint, the paper underscores the importance of mechanisms like green bonds, supportive regulation, and corporate culture in driving sustainable M&A practices. Its clear structure, strong evidence base, and forward-looking recommendations make it a valuable resource for both researchers and practitioners. While minor improvements such as updating citations and enhancing the conclusion could increase its effectiveness, the paper stands as a timely and authoritative contribution to the field of sustainable finance.

Are the rationale for, and objectives of, the Systematic Review clearly stated?

Yes

Is the statistical analysis and its interpretation appropriate?

Yes

If this is a Living Systematic Review, is the ‘living’ method appropriate and is the search schedule clearly defined and justified? (‘Living Systematic Review’ or a variation of this term should be included in the title.)

Yes

Are sufficient details of the methods and analysis provided to allow replication by others?

Yes

Are the conclusions drawn adequately supported by the results presented in the review?

Yes

Reviewer Expertise:

Sustainable Finance, sustainability, M&A., Finance

I confirm that I have read this submission and believe that I have an appropriate level of expertise to confirm that it is of an acceptable scientific standard.

# Reviewer report: Reviewer response for version 1

**DOI:** 10.5256/f1000research.164752.r373246

**Published:** 2025-04-29

**Authors:** Saurabh Kumar Srivastava

**Affiliations:** 1 Bennett University, Noida, Uttar Pradesh, India

This manuscript provides a comprehensive and systematic review of sustainable finance in mergers and acquisitions (M&A), offering valuable insights into the evolving intersection of environmental, social, and governance (ESG) factors with corporate transactions. The study stands out for its rigorous methodology, employing bibliometric analysis and machine learning to evaluate 41 key publications from Web of Science and Scopus. It effectively maps research trends, highlighting the growing focus on sustainability post-2016, with China and Australia leading in contributions. The paper identifies critical gaps, such as the underrepresentation of emerging markets and the need for deeper exploration of greenwashing, AI applications, and cross-border M&A dynamics.

 The review excels in synthesizing diverse theoretical perspectives, including stakeholder and shareholder theories, while presenting empirical evidence on how ESG alignment enhances M&A outcomes. Its structured approach—categorizing research into bidder vs. target CSR comparisons, ESG’s impact on deal performance, and post-M&A sustainability effects—provides clarity and actionable insights. The inclusion of temporal word clouds and keyword co-occurrence analysis enriches the discussion, revealing shifts from financial metrics to broader ESG determinants.

 Practical implications are robust, emphasizing the role of green bonds, regulatory frameworks, and corporate culture in sustainable M&A. The manuscript’s clear writing, well-supported conclusions, and forward-looking research questions make it a significant contribution to academia and industry. Minor enhancements, such as updating references and refining the conclusion, would further strengthen its impact. Overall, this review is a timely and authoritative resource for advancing sustainable finance research.

Are the rationale for, and objectives of, the Systematic Review clearly stated?

Yes

Is the statistical analysis and its interpretation appropriate?

Yes

If this is a Living Systematic Review, is the ‘living’ method appropriate and is the search schedule clearly defined and justified? (‘Living Systematic Review’ or a variation of this term should be included in the title.)

Yes

Are sufficient details of the methods and analysis provided to allow replication by others?

Yes

Are the conclusions drawn adequately supported by the results presented in the review?

Yes

Reviewer Expertise:

Consumer behaviour, Entrepreneurship, Marketing

I confirm that I have read this submission and believe that I have an appropriate level of expertise to confirm that it is of an acceptable scientific standard.

# Reviewer report: Reviewer response for version 1

**DOI:** 10.5256/f1000research.164752.r373240

**Published:** 2025-04-03

**Authors:** Abhishek Sinha

**Affiliations:** 1 IBS Hyderabad, Hyderabad, India

**Summary of the Manuscript** Sustainable Development Goals were introduced in 2016, sustainable finance has become an increasingly important topic in merger and acquisition research. Research is progressing at a rapid pace in China and Australia, while emerging nations are falling behind. This study specifies the necessity of conducting a more comprehensive examination of the habits of company representatives regarding sustainable investments. This aspect has been neglected in earlier research. Additionally, because global M&As are becoming more common, research on cross-border M&As must take precedence over domestic ones. Finally, there is a gap in the current body of knowledge about greenwashing, green audits, and the use of AI in sustainable finance

**Originality and Contribution to the Field:**

 Is the research original? Reviewer: Yes.

 Does it make significant contribution to the field? Reviewer: This part may be improved slightly with more justifications.

 Is the research question well framed? Reviewer: Yes.

**Quality of the Research**

 Is the methodology strong? Reviewer: Yes

 Are the data collection and analysis methods appropriate and clearly explained? Reviewer: yes.

 Are the results presented clearly and accurately? Reviewer: Yes.

**Clarity and Structure**

 Is the paper well-organized and logically structured? Reviewer: Yes

 Are the arguments presented clearly and coherently? Reviewer: Yes

 Are there any issues with the readability of the paper? Reviewer: The language of the paper is clear, however, draft needs a proper proofread before the next submission.

**Literature Review and Reference**  

 Does the manuscript provide a comprehensive and up-to-date literature review?      Reviewer: Authors are advised to add few more updated reviews of recent indexed works to make this section more significant.

 Are the references relevant, sufficient, and properly cited?      Reviewer: Yes.

**Conclusion and Implications**  

 Are the conclusions supported by the findings? Reviewer: yes.

 Are the implications for future research and practice clearly discussed? Reviewer: Though the future research directions are presented properly, the authors can have a second look to find out if something more can be added, especially in relation to AI. 

**Recommendation**

 Research methods are appropriate and robust. The interpretation of results is logical and tied directly to the research.

 The literature review should be comprehensive and relevant. However, a few more temporal and contemporary studies should be added to make it up-to-date.

 The conclusion should logically follow from the findings and offer insights into the broader implications of the research. The conclusion can be compact.

 The author may use a professional editing service to improve readability.

**Minor Revision** – The manuscript requires minor revisions before indexing.

**Recommended Decision: **Minor Revision

Are the rationale for, and objectives of, the Systematic Review clearly stated?

Yes

Is the statistical analysis and its interpretation appropriate?

Yes

If this is a Living Systematic Review, is the ‘living’ method appropriate and is the search schedule clearly defined and justified? (‘Living Systematic Review’ or a variation of this term should be included in the title.)

Yes

Are sufficient details of the methods and analysis provided to allow replication by others?

Yes

Are the conclusions drawn adequately supported by the results presented in the review?

Yes

Reviewer Expertise:

ESG, Sustainability, IPO Underpricing

I confirm that I have read this submission and believe that I have an appropriate level of expertise to confirm that it is of an acceptable scientific standard.
